# Dynamic prefrontal coupling coordinates adaptive decision-making

**DOI:** 10.21203/rs.3.rs-6296852/v1

**Published:** 2025-04-09

**Authors:** Xinyuan Yan, Seth D. König, R Becket. Ebitz, Benjamin Y. Hayden, David P. Darrow, Alexander B. Herman

**Affiliations:** 1 Department of Psychiatry, University of Minnesota; Minneapolis, MN, USA.; 2 Department of Neurosurgery, University of Minnesota; Minneapolis, MN, USA.; 3 Department of Neurosurgery, Baylor College of Medicine, Houston, TX, USA.; 4 Department of Neuroscience, Universite de Montreal, Montreal, Quebec, Canada

**Keywords:** adaptation, value, uncertainty, stay, switch, dorsolateral prefrontal cortex, dorsomedial prefrontal cortex, inter-regional coupling

## Abstract

Adaptive decision-making requires flexibly maintaining or changing behavior in response to uncertainty. While the dorsomedial (dmPFC) and dorsolateral (dIPFC) prefrontal cortex are each essential for this ability, how they coordinate to drive adaptation remains unknown. Using intracranial EEG recordings from human participants performing a dynamic reward task, we identified distinct, frequency-specific computations: dmPFC high-gamma activity encoded uncertainty before stay decisions but transitioned to prediction error before switches, while theta activity shifted from uncertainty to value representation. In contrast, dIPFC theta activity signaled both value and uncertainty before stays, but predominantly value before switches. Crucially, these regions coordinated through two temporally specific coupling mechanisms that predicted behavioral changes: theta-theta amplitude coupling during feedback processing and theta-gamma phase coupling before decisions. Both coupling mechanisms strengthened before switches, suggesting that changing behavior requires greater dmPFC-dIPFC integration than maintaining. These findings reveal how the dorsal prefrontal cortex employs frequency-specific computations and precise temporal coordination to guide adaptive behavior.

## Introduction

The ability to process feedback and adapt behavior is fundamental to survival across species. This mechanism operates in foraging animals and humans alike, enabling effective learning, planning and problem-solving. A critical challenge is determining when to maintain current choices versus when to switch to alternatives based on feedback. While previous research suggests that relative value and relative uncertainty play important roles in such decisions^1–3^, how these variables are computed remains poorly understood. Unraveling this dynamic evaluation process is crucial not only for explaining adaptive behavior but also for understanding maladaptive patterns in neuropsychiatric disorders^4,5^, where disrupted value-uncertainty processing may drive symptoms^3^.

Dorsomedial prefrontal cortex (dmPFC) and dorsolateral prefrontal cortex (dIPFC) play distinct yet complementary roles in adaptive decision-making (Blanchard & Gershman, 2018; Sazhin et al., 2024). The dmPFC has been implicated in a variety of functions, including detecting uncertainty^6^, evaluating the need for cognitive control^7^, and signaling the value of exploring versus exploiting current options^8^. The dmPFC appears to be central in goal-directed behavior, with lesions to this area resulting in decreased motivation to act^9^, while stimulation can induce an “urge” to act towards a goal^10,11^. In contrast, the dIPFC is more directly responsible for implementing cognitive control and action selection^7,12^.

The dmPFC and dIPFC interact dynamically to guide adaptive decision-making, with each region playing distinct but coordinated roles^13,14^. The dmPFC monitors performance, detects uncertainty, and integrates feedback, shaping the dIPFC’s role in selecting and executing actions. This interaction follows a distinct temporal pattern: the dmPFC is more active after feedback, evaluating outcomes and updating expectations, while the dIPFC is engaged during decision-making, preparing and selecting the next action^15^.

Based on these distinct roles, we hypothesized that dmPFC and dIPFC form an integrated system for adaptive behavior: the dmPFC detects and signals information for adaptation upon feedback, while the dIPFC implements the resulting behavioral changes.

**Fig. 1.**
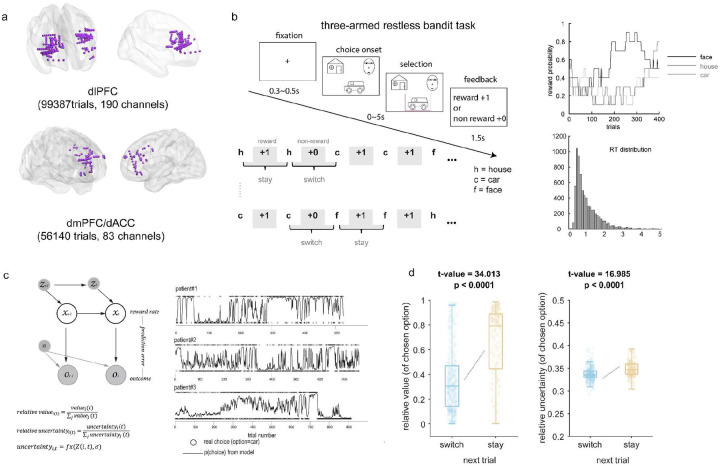
Experimental setup, task design, computational modeling, and behavioral results. (a) Intracranial EEG recordings from 14 epilepsy patients, with 190 channels in the dorsolateral prefrontal cortex (dIPFC) and 83 channels in the dorsomedial prefrontal cortex (dmPFC). (b) Three-armed restless bandit task. Patients chose one of three options (face, car, house), followed by reward or no-reward feedback. Upper right panel: example trials showing how reward probability for each bandit varied in a random walk manner (on each correct trial, there was a 10% chance that the reward probability for each target would either increase or decrease by 0.2, with these probabilities bounded between 0.1 and 0.9, see Methods). Lower right panel: Response time (RT) distribution across all patients (model-free results and more time information see TableS2 & Table S3). (c) Volatile Kalman filter model implementation and model fits. The model computes trial-by-trial value and uncertainty estimates for each arm. Relative value (RV) and relative uncertainty (RU) were calculated as the ratio of the chosen option’s value/uncertainty to the sum across all options. Right panels show model fits from three representative patients: colored dots indicate actual choices for specific options (e.g., car), black lines show the model-predicted choice probabilities. Bayesian model comparison results revealed that the volatile Kalman filter with relative value and relative uncertainty incorporated into the softmax function outperformed other models (model validation analyses see Supplementary Notes1, Fig. S1, model comparison results see Table S4) (d) Choice behavior of the next trial as a function of the current chosen option’s relative value and uncertainty. Left box plots showing the distributions of relative value and right box showing uncertainty for stay versus switch trials. Higher relative value and uncertainty promote stay decisions, while lower relative value and uncertainty lead to switches (n=10 points/condition shown using stratified sampling for visualization purposes, statistical significance assessed using linear mixed-effects models across all trials, see Methods).

To test this hypothesis, we analyzed intracranial EEG recordings from dorsolateral prefrontal cortex (dIPFC, 190 channels, 7191 unique trials across participants, 99387 trials across all channels) and dorsomedial prefrontal cortex (dmPFC, 83 channels, 6131 unique trials, 56140 trials across all channels) in 14 participants with drug-resistant focal epilepsy (Fig. 1a and Table S1) during an adaptive task. The electrode placement was determined solely by clinical needs in these patients. Participants performed a three-armed restless bandit task (Fig. 1b) simulating real-world decision-making with dynamic, probabilistic rewards^16^. We investigated how these key prefrontal regions dynamically represent and integrate value and uncertainty information to guide decisions about maintaining versus changing behavior. We applied a series of Kalman filter models^17,18^ (Fig. 1c) utilizing hierarchical Bayesian inference (HBI)^19^ to fit our behavioral data and selecting the winning model to estimate single trial value and uncertainty, modeling the dynamic decision-making process (Table S4).

Our findings revealed a dynamic prefrontal mechanism for adaptive decision-making. Behaviorally, participants were more likely to switch choices when an alternative offered higher expected value and greater uncertainty (Fig. 1d). The neural data showed that dmPFC and dIPFC employed distinct, frequency-specific computations that varied between stay and switch decisions. Critically, coordination between these regions occurred through two temporally specific coupling mechanisms: theta-theta amplitude coupling during post-feedback processing and theta-gamma phase coupling during the pre-decision period. Both coupling mechanisms strengthened before switches, with post-feedback-stage coupling correlating with prediction errors and uncertainty, while pre-decision coupling tracked prediction errors and value. These findings illuminate how the prefrontal cortex coordinates frequency-specific computations and temporal dynamics to guide adaptive behavior.

## Results

### Value and uncertainty jointly determine behavioral persistence versus change

Behavioral results revealed a systematic relationship between participants’ choices and the dynamic properties of chosen options. When faced with probabilistic rewards that evolved over time, participants’ decisions to maintain or change their behavior depended on both the relative value and relative uncertainty of their chosen option. Specifically, participants were more likely to persist with their current choice when it offered both higher relative value (t=34.013, p<0.0001) and greater relative uncertainty compared to alternatives (t=16.985, p<0.0001). Conversely, when a chosen option had lower relative value and uncertainty, participants were more likely to abandon it and switch to a different alternative (Fig. 1d). This pattern suggests that behavior was influenced by a desire to both maximize reward and minimize uncertainty, with individuals integrating information about value and uncertainty when determining whether to maintain or change behavior.

### Differential roles of dIPFC and dmPFC in feedback and decision stages

**Fig. 2.**
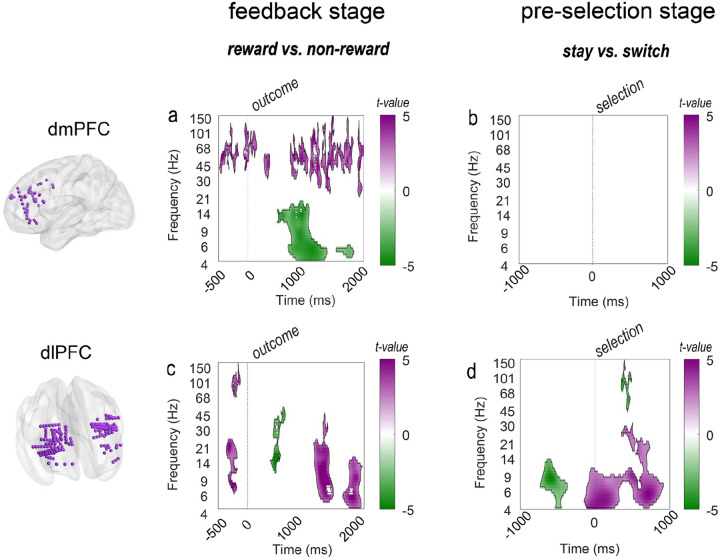
Differential roles of dIPFC and dmPFC in feedback processing and choice selection. (a, c) Time-frequency analyses (t-value maps) of local field potentials in the dIPFC and dmPFC during post-feedback processing (2000ms after outcome onset). Both regions show significant differences in neural activity between reward and non-reward feedback conditions. (b, d) The dIPFC exhibits distinct activity patterns associated with stay and switch decisions before selection, while the dmPFC shows no significant differences between these decision types.

We analyzed neural oscillations in dmPFC and dIPFC during two critical task periods: the post-feedback stage (2000ms after outcome onset), when value and uncertainty estimates are updated, and the pre-selection stage (1000ms before the next choice), when those estimates are transformed into choice. Time-frequency analyses (Methods) revealed a clear regional dissociation. The dmPFC was primarily active during the post-feedback stage (Fig. 2a) and, strikingly, showed no encoding of stay versus switch choices (Fig. 2b & Supplementary Notes 2, Fig. S2). In contrast, the dIPFC exhibited dual functionality, processing reward information during post-feedback (Fig. 2c) and distinctly encoding stay versus switch choices during the pre-selection stage (Fig. 2d).

Prior to feedback, high-frequency activity (>30Hz) in dmPFC and theta-alpha power (4–9 Hz) in dIPFC was greater on rewarded compared to unrewarded trials. Since reward probabilities in this task change slowly, they were somewhat predictable, suggesting that this pre-feedback activity (500ms before the outcome onset) may reflect anticipatory signals that have been observed in these frequency bands^20^. Consistent with this anticipatory processing, feedback responses revealed distinct spectral signatures across regions. dIPFC exhibited stronger low-frequency (4–14 Hz) activity for reward feedback, peaking around 1500 ms post-feedback. In contrast, activity in dmPFC dissociated between frequency bands: high-frequency (>30Hz) activity increased for rewards while low-frequency (4–14 Hz) activity increased for non-rewards, suggesting a frequency-specific mechanism for win and loss processing.

These findings reveal a fundamental dissociation: the dmPFC primarily processes feedback (reward or non-reward), while the dIPFC additionally signals subsequent behavioral changes. This distinction raises the question of how these regions process specific computational variables that drive behavioral changes. To address this, we focused our subsequent analyses on the high-gamma (70–150 Hz) and theta (4–9 Hz) bands. This targeted approach was motivated by several factors. First, high-gamma activity is a well-established proxy for local neural activity and has been consistently implicated in cognitive processing (e.g., feedback and learning signal processing)^21,22^. Second, theta oscillations are critical in action execution^23^ and inter-area communication^24^. Moreover, these frequency bands demonstrated the strongest and most consistent task-related effects in our initial time-frequency analysis. With these frequency bands identified, we next investigated how these regions represent prediction errors, a key computational signal that drives learning, value updating, and behavioral changes^25^.

Color scales represent t-values, with deeper purple colors indicating higher t-values. Significant clusters (defined by the cluster-based permutation tests, permutation 5000 times, p<0.05) are outlined in black. Time 0 represents feedback onset in (a, c) and selection (i.e., decision has been made) onset in (b,d). Frequency bands (logarithmically spaced) are displayed on the y-axis, ranging from 4 Hz to 150Hz. Statistical analyses were performed using linear mixed-effects models (LMEs) with patient ID and channel ID as random effects (decision coding: stay = 1, switch = 0; feedback coding: reward = 1, non-reward = 0).

### Neural representation of prediction error shows choice-dependent patterns

**Fig. 3:**
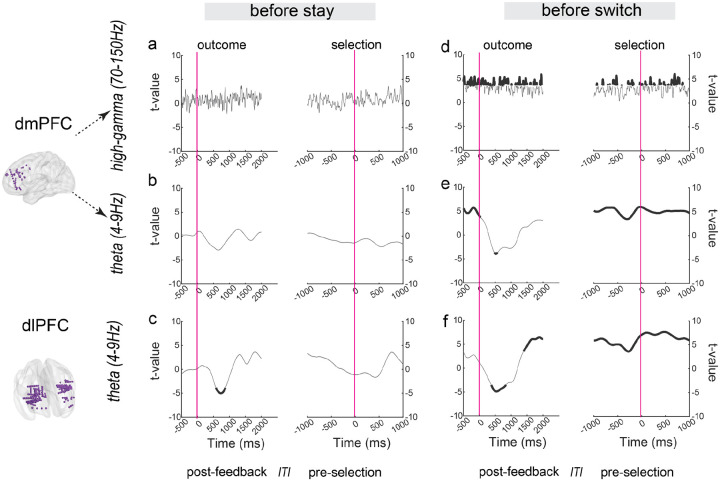
Neural representations of prediction error (PE) in dIPFC and dmPFC preceding stay/switch decisions (a-c) PE representation preceding stay decisions: (a) dmPFC high-gamma band (70–150 Hz), (b) dmPFC theta band (4–9 Hz), and (c) dIPFC theta band showing minimal PE representation, with only a brief significant window in dIPFC. (d-f) PE representation preceding switch decisions: (d) dmPFC high-gamma band, (e) dmPFC theta band, and (f) dIPFC theta band, all showing robust PE representation.

Analysis of prediction error (PE) representation revealed a striking dissociation between trials preceding stay versus switch decisions (neural correlates for PE based on all trials see Fig. S3). During trials that preceded *switch* decisions, both regions showed robust PE representation: dmPFC exhibited strong PE signals in both high-gamma and theta bands (Fig. 3d–e), while dIPFC showed reliable PE representation in the theta band (Fig. 3f). However, during trials preceding *stay* decisions, PE representation was notably absent in both regions (Fig. 3a–b), with dIPFC showing only a brief window of significance (~300 ms) in post-feedback stage (Fig. 3c). This pattern suggests that PE representation plays a critical role in driving behavioral adaptation.

Lines represent t-values from linear mixed-effects regression (LME), with shaded areas indicating SEM from LME; smaller SEM indicates more precise estimation. Black segments of the lines indicate periods with significant prediction error representation (p < 0.001, FDR-BH correction). Time 0 represents outcome onset (left) and subsequent selection onset (right). The inter-trial interval (ITI) is the period between the end of one trial and the beginning of the next trial (mean±SD = 0.626s±0.072s)

### Neural representation for value and uncertainty shows choice-dependent patterns

**Fig. 4:**
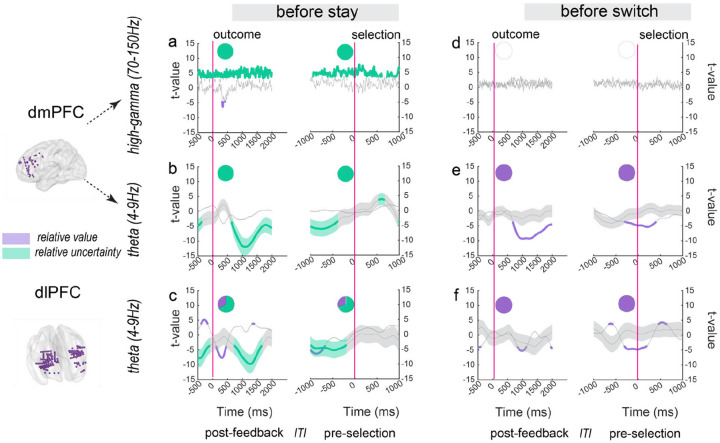
Choice-dependent neural representation of value and uncertainty in prefrontal regions Neural representations of relative value (RV) and relative uncertainty (RU) during stay and switch decisions. (a-c) Neural activity preceding stay decisions: (a) dmPFC high-gamma band (70–150 Hz) and (b) dmPFC theta band (4–9 Hz) exclusively represent relative uncertainty, while (c) dIPFC theta band represents both variables with predominant uncertainty representation. (d-f) Neural activity preceding switch decisions: (d) dmPFC high-gamma shows no significant representation, (e) dmPFC theta band selectively represents relative value, and (f) dIPFC theta band represents also selectively represents value.

Since PE correlated strongly with relative value (RV; r = 0.445, p < 0.00001) but not with relative uncertainty (RU; r = 0.0005, p = 0.961), we hypothesized an analogous pattern of neural activity for representing relative value, stronger representation before switch trials. However, given our behavioral findings that uncertainty drives stay behavior, we expected relative uncertainty representations might show the opposite pattern, with robust representation specifically preceding stay trials. To answer this question, we analyzed how these regions signal relative value and uncertainty (Methods). dmPFC high-gamma activity showed selective representation of relative uncertainty (RU) but not relative value (RV) across all trials (Fig. S4). This selectivity was maintained in trials preceding stay decisions, with dmPFC high-gamma and theta bands tracking RU, while dIPFC theta activity encoded both RV and RU, with RU dominating. In contrast, a different pattern emerged before switch decisions: dmPFC high-gamma activity no longer encoded either variable, while dmPFC and dIPFC theta bands primarily tracked RV (Fig. 4).

Time courses show relative value (purple) and relative uncertainty (green) representation strengths measured by t-values from linear mixed-effects regression, with shaded areas indicating SEM from LME, smaller SEM indicates more precise estimation. Colored segments indicate significant representation periods (p < 0.001, FDR-BH correction). Time is aligned to outcome onset (left) and next selection onset (right). Inset pie charts quantify the proportion of significant time points for relative value versus relative uncertainty representation in each condition. The inter-trial interval (ITI) is the period between the end of one trial and the beginning of the next trial (mean±SD = 0.626s±0.072s)

### dmPFC-dIPFC communication predicts behavioral adaptation

Further analyses of neural activity patterns revealed intriguing similarities in theta-band oscillations between dIPFC and dmPFC despite their distinct computational roles in decision-making. Both regions exhibited parallel temporal dynamics in theta-band prediction error signalling preceding switch decisions (Fig. 3d–f) and showed coordinated shifts in value and uncertainty representations across decision types (Fig. 4). This observation led us to ask a fundamental question: how do these prefrontal regions communicate to orchestrate their complementary functions during decision-making? To address this question, we examined the coupling between dmPFC and dIPFC during post-feedback and pre-selection periods and investigated how these coupling patterns predict subsequent behavioral adaptation.

The parallel theta-band dynamics we observed between regions, combined with theta’s established role in long-range neural communication^26,27^, led us first to examine theta-theta amplitude-amplitude coupling (AAC, Methods). Additionally, while the temporal patterns of dmPFC high-gamma activity differed from dIPFC theta activity, these distinct patterns suggested another potential coordination mechanism. Given that high gamma reflects local neural computations^28^ and that phase-amplitude coupling (PAC, Methods & Fig. S5) coordinates information flow between brain regions during cognitive processing^29,30^, we examined whether the dmPFC high gamma amplitude fluctuation might be organized according to the dIPFC theta phase. Examining inter-regional PAC could provide evidence for a communication pathway between dmPFC and dIPFC for integrating value and uncertainty computations during decision-making.

**Fig.5.**
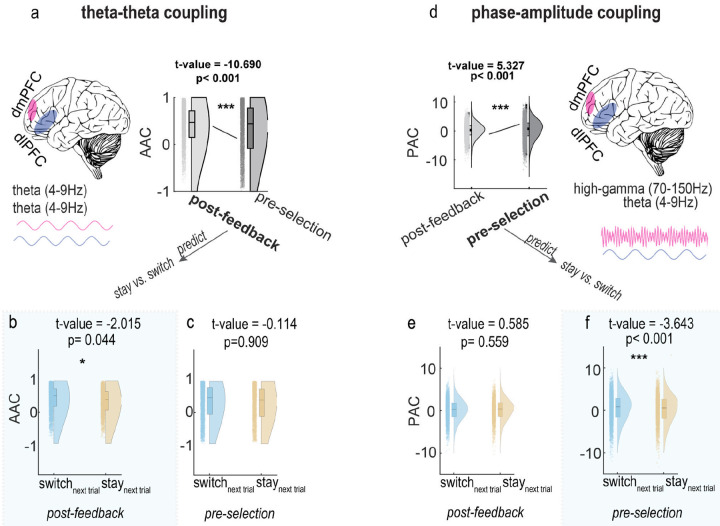
Dynamic inter-regional coupling between dmPFC and dIPFC predicts decision changes Left panel: theta-theta amplitude-amplitude coupling (AAC) (a) Comparison of AAC strength between post-feedback and pre-selection stages, showing stronger coupling during post-feedback period (b) Post-feedback stage AAC significantly predicts subsequent switch decisions (higher AAC associated with increased switch probability) (c) Pre-selection stage AAC shows no significant predictive relationship with subsequent decisions. Right panel: phase-amplitude coupling (PAC) between dIPFC theta phase and dmPFC high-gamma amplitude (d) Comparison of PAC strength between stages, showing stronger coupling during pre-selection stage (e) post-feedback stage PAC shows no significant predictive relationship with subsequent decisions (f) Pre-selection stage PAC significantly predicts subsequent switch decisions (higher PAC associated with increased switch probability).

Analysis of these coupling mechanisms revealed two distinct patterns that predicted subsequent behavioral choices (Fig. 5). First, theta-theta amplitude coupling between dmPFC and dIPFC was stronger during the post-feedback stage than the pre-selection stage (AAC_post-feedback_ Vs. AAC_pre-selection_, t=−10.690, p<0.001, stage coding in LMEs: pre-selection = 1, post-feedback = 0) and the post-feedback AAC marginally predicted subsequent switch decisions (t=−2.015, p=0.044; decision coding in LMEs: stay = 1, switch = 0; higher AAC_post-feedback_ associated with increased switch probability). Second, phase-amplitude coupling between dIPFC theta and dmPFC high gamma was elevated during the pre-selection stage (PAC_post-feedback_ vs. PAC_pre-selection_, t=5.327, p<0.001), and the pre-selection PAC predicted switch decisions (t=−3.643, p<0.001, higher PAC_pre-selection_ associated with increased switch probability)

Statistical significance: * p<0.05, ** p<0.01, *** p<0.001. All statistics derived from linear mixed-effects regression analysis (LMEs). LMEs with patient ID as random effect were used to analyze dIPFC-dmPFC coupling (stage coding: pre-selection = 1, post-feedback = 0) and coupling’s predictive value for subsequent decisions (stay = 1, switch = 0)

**Figure 6.**
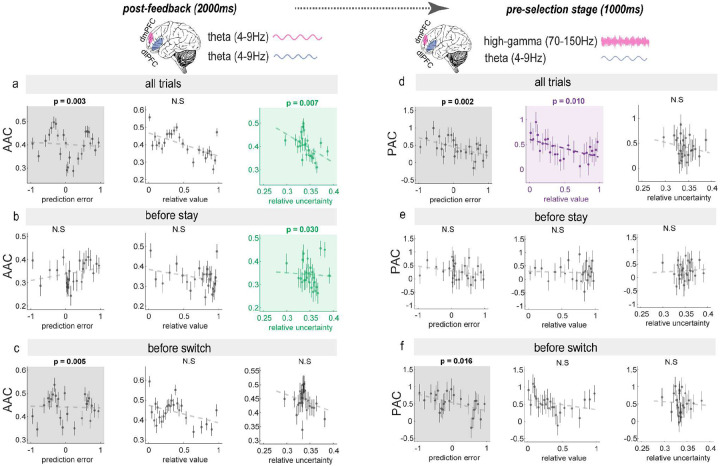
Decision-specific patterns of inter-regional coupling and their relationships with computational variables Analysis of coupling-variable relationships during post-feedback and pre-selection periods across different decision contexts. Left panels, post-feedback stage amplitu-deamplitude coupling (AAC) (a) Combined trials: AAC exhibits significant negative correlations with both prediction error (PE) and relative uncertainty (RU) (b) Stay-decision trials: AAC shows selective negative correlation with RU (c) Switch-decision trials: AAC shows selective negative correlation with PE. Right panels: the pre-selection stage phase-amplitude coupling (PAC) (d) Combined trials: PAC shows significant negative correlations with both PE and relative value (RV) (e) Stay-decision trials: No significant correlations observed (f) Switch-decision trials: PAC exhibits selective negative correlation with PE.

These temporally specific coupling patterns, which predict behavioral changes, raised a key question: What information was conveyed between regions during these distinct coupling modes? To address this, we examined how post-feedback AAC and pre-selection stage PAC correlate with our computational variables (prediction error, relative value, and relative uncertainty) during trials leading to different decisions. Intriguingly, these two coupling mechanisms exhibited distinct temporal relationships with computational variables. During post-feedback processing, where the AAC could significantly predict subsequent behaviors), AAC_post-feedback_ was negatively correlated with both PE and relative uncertainty (Fig.6a) across all trials, with a choice-dependent dissociation; it was specifically negatively correlated with relative uncertainty before stay decisions (Fig. 6b) and with PE before switch decisions (Fig. 6c). During the pre-selection period, where the PAC could significantly predict subsequent behaviors, PAC_pre-selection_ showed a different pattern; while correlated negatively with both PE and relative value across all trials (Fig.6d), these correlations were only significant before switch decisions not stay decisions (Fig.6e), where PAC was specifically negatively correlated with PE (Fig.6f).

The strength of both coupling mechanisms predicted behavioral adaptation: Stronger theta-theta AAC during post-feedback and stronger theta phase-high gamma PAC during pre-selection were associated with an increased likelihood of switch decisions. Conversely, weaker coupling between these regions was associated with stay decisions, suggesting that reduced inter-regional communication might reflect a maintenance of the current behavioral strategy when the existing choice policy is deemed appropriate.

Together, these findings reveal an elegant mechanism for information transfer between prefrontal regions during adaptive decision-making. The temporal specificity of theta-theta AAC and theta phase-high gamma PAC suggests these are the primary mechanisms through which dmPFC and dIPFC coordinate their activity to integrate computational variables and guide behavioral adaptation. The increased coupling strength associated with switch decisions indicates that stronger inter-regional communication is particularly crucial when updating behavioral strategies. In contrast, reduced coupling may support the maintenance of established choice patterns during stay decisions.

Data points represent mean coupling values (AAC or PAC) for data binned by percentiles (25 bins), with error bars indicating the standard error of the mean (SEM) for each percentile bin. Statistical significance levels: * p<0.05, ** p<0.01, *** p<0.001, derived from linear mixed-effects models (LMEs). LMEs with patient ID as random effect were used to analyze dIPFC-dmPFC coupling’s relationship with PE, relative value, and relative uncertainty.

## Discussion

Our study investigated the neural mechanisms underlying adaptive decision-making in dynamic environments, revealing how the human prefrontal cortex processes feedback and integrates value and uncertainty information to guide behavioral changes. By analyzing intracranial EEG recordings from dorsomedial prefrontal cortex (dmPFC) and dorsolateral prefrontal cortex (dIPFC) during a three-armed restless bandit task and applying a volatile Kalman filter model, we uncovered a sophisticated, multi-stage process in which these regions coordinate their activities to drive stay/switch decisions.

### A dynamic prefrontal system for processing feedback and guiding decisions

Our findings demonstrate that dmPFC and dIPFC form a dynamic circuit with distinct yet coordinated roles in processing feedback and guiding subsequent decisions. A striking dissociation emerged: the dmPFC showed robust responses to feedback (reward versus non-reward) but critically, did not directly encode stay versus switch choices^31^. In contrast, the dIPFC exhibited dual functionality, processing reward information and distinctly encoding subsequent choice behavior. This fundamental dissociation suggests a functional division where the dmPFC serves primarily as a computational hub for feedback processing, while the dIPFC may translate these computations into behavioral decisions.

The dmPFC showed remarkable flexibility in its encoding patterns depending on upcoming choices. Before stay decisions, high-gamma and theta activity in dmPFC selectively represented relative uncertainty while showing minimal value or prediction error encoding. This selective uncertainty encoding aligns with our behavioral finding that high uncertainty promotes staying with the current option, suggesting the dmPFC might signal “stay and gather more information” when uncertainty is high.

This pattern shifted dramatically before switch decisions in a frequency-specific manner. In the high-gamma band, dmPFC activity transitioned from selective uncertainty representation to robust prediction error representation. Meanwhile, in the theta band, dmPFC activity shifted from encoding selective uncertainty before stay decisions to representing relative value before switches, with both frequency bands showing strong prediction error encoding before behavioral changes (switch trials). This coordinated representation aligns with the computational architecture of learning: prediction errors directly influence value updating, while uncertainty modulates learning rate^32^. These frequency-specific transformations, from uncertainty to prediction error in high-gamma, and from uncertainty to value in theta band, suggest that dmPFC employs distinct frequency pathways to process different aspects of feedback that guide subsequent decisions.

The dIPFC exhibited complementary but distinct patterns of choice-dependent encoding. Before stay decisions, theta activity represented both value and uncertainty, with uncertainty representation predominating. This pattern suggests that dIPFC integrates multiple information streams to support maintaining current behavior. However, before switch decisions, dIPFC theta shifted to predominantly signaling relative value while also showing robust prediction error representation. This pattern suggests a reprioritization of information when behavioral changes are needed, aligning with previous findings of prefrontal mechanisms of adaptive control^13,15^.

### Temporally specific inter-regional communication prepared for subsequent behaviors through distinct coupling mechanisms

A key finding of our study is that the dmPFC and dIPFC communicate through distinct coupling mechanisms that are temporally specific and predictive of behavioral adaptation. These mechanisms are not merely generic measures of connectivity but instead appear to convey specific computational information at different stages of the decision-making process. During the post-feedback stage, dmPFC and dIPFC interacted through theta-theta amplitude coupling (AAC), aligning closely with the regional patterns we observed. When feedback indicated an outcome worse than expected (a more negative prediction error) or when uncertainty was resolved, stronger AAC_post-feedback_ emerged, suggesting that the system was preparing for behavioral change. Moreover, this relationship became even more specific based on upcoming decisions, mirroring the regional specialization we observed: before stay decisions, AAC_post-feedback_ selectively correlated with relative uncertainty, matching the uncertainty-dominated encoding in both regions’ local activity (Fig. 4a–c).

In contrast, AAC_post-feedback_ selectively correlated with prediction error before switch decisions, paralleling the strong prediction error representation we observed in both regions’ local activity (Fig. 3d–f). During the pre-selection period, the coupling mechanism shifted to dIPFC theta-dmPFC gamma phase-amplitude coupling (PAC). This shift in coupling mode aligns with the transformation we observed in regional representation patterns (Fig. 3–4), from uncertainty-dominated to value/prediction-error-dominated processing. Stronger PAC_pre-selection_ was associated with more negative prediction errors and lower relative values, particularly before switch decisions.

These results suggest that stronger dIPFC-dmPFC coupling, both AAC during post-feedback stage and PAC before selection, reflects the integration of information favoring a change in behavior. When outcomes are worse than expected, values and uncertainties of alternatives are relatively low, and increased coordination between regions may drive the transition from the current option to alternatives. Conversely, the weaker coupling we observed with stay decisions suggests that reduced inter-regional coupling may reflect a maintenance of the current behavioral strategy when the existing choice policy is deemed appropriate.

### Hierarchical processing of computational variables

Our findings suggest a hierarchical processing structure within the prefrontal cortex for adaptive decision-making^33,34^. While the dmPFC itself does not appear to directly encode decisions (stay vs. switch; Fig. 2), it processes the computational variables that guide behavior and may communicate these to the dIPFC through two distinct coupling mechanisms. The dmPFC’s computational signals influence behavior through both theta-theta AAC in post-feedback evaluation and theta-gamma PAC during action selection. Specifically, the dmPFC’s uncertainty and prediction error signals are first processed through AAC during post-feedback. Then its high-gamma-related computations (monitoring the uncertainty before stay but PE before switch) influence final choice implementation through PAC with the dIPFC theta. This dual-coupling architecture suggests that the dmPFC serves primarily as a computational hub, processing decision-relevant variables^35,36^, while the dIPFC functions more as an action selection mechanism^37^, translating these computations into behavioral choices akin to a softmax function operating on the outputs of the dmPFC. This temporal organization, where uncertainty assessment precedes value processing in prefrontal coupling dynamics, enables a systematic evaluation process that culminates in value-based action selection.

### Limitations

This study has several limitations. First, while intracranial recordings provide unique temporal and spatial precision, our findings are correlational. Causal manipulation techniques would be necessary to establish the necessity and sufficiency of these frequency-specific coupling mechanisms for adaptive decision-making. Second, our analyses focused on dmPFC-dIPFC interactions, but adaptive decision-making likely involves broader networks, including the striatum and OFC^38^. Understanding how our observed coupling mechanisms interact with these broader circuits represents an important next step.

### Clinical relevance and future directions

Disruptions in value and uncertainty processing are implicated in a range of neuropsychiatric disorders^3,5^. Our findings provide a specific neural mechanism that the dynamic interplay between dmPFC and dIPFC through temporally specific coupling could be disrupted in these conditions. In major depressive disorder, where patients show behavioral inertia^39^, and in obsessive-compulsive disorder, characterized by abnormal habitual tendencies^40,41^, strengthening dIPFC-dmPFC coupling during action selection might facilitate the translation of uncertainty and value computations into behavioral changes. Future research should investigate how to modulate these coupling mechanisms in clinical populations effectively.

## Online Methods

### Ethics approval

The experiment’s procedures were by the standards set by the Declaration of Helsinki and approved by the local Research Ethics Committee of the University of Minnesota, Twin Cities. Participants provided written informed consent after the experimental procedure had been fully explained and were reminded of their right to withdraw at any time during the study.

### Participants

In the current study, we recorded directly from a total of 190 implanted sites in the dIPFC (Fig. 1a) and 83 implanted sites in the dmPFC in 14 patients (mean age, 39.93 ± 11.45; range, [25, 61]; 4 female and 10 male; Table S1). All patients had refractory epilepsy, and they volunteered for this study and provided their informed consent. The participants performed the three-armed restless bandit task while they stayed at the hospital and were implanted with electrodes for seizure monitoring. The locations of the implanted electrodes were determined solely by clinical needs. No statistical methods were used to predetermine sample sizes.

### Three-armed restless bandit task

Participants were free to choose between three targets for the potential to earn a reward of 1 point. Each target is associated with a hidden reward probability that randomly and independently changes throughout the task. We seeded each participant’s reward probability randomly to prevent biases due to particular kinds of environments. Specifically, on each correct trial, there was a 10% chance that the reward probability for each target would either increase or decrease by 0.2, with these probabilities bounded between 0.1 and 0.9. Due to the variable and independent nature of the rewards, participants could only estimate the probabilities by actively sampling from the targets and accumulating their reward experiences over time. Trials were excluded if participants failed to respond within 5 seconds. The task was programmed in MATLAB using Psychtoolbox 3 (http://psychtoolbox.org/). Stimuli were presented on a monitor positioned approximately 60 cm from the participant’s face and centered in their field of view while they sat upright in their hospital bed.

### Visualization for Figure 1F

For visualization of the behavioral results, we employed box plots with overlaid data points to show the distribution of relative value and relative uncertainty between stay and switch trials. The box plots display the median (central line), first and third quartiles (box edges), and the full range of the data (whiskers). To maintain visual clarity while representing the full distribution, we used a stratified subsampling approach to display individual data points. Specifically, we sampled 10 points per condition using a binned sampling method that preserves the underlying distribution: the data was first sorted and divided into bins, with one point randomly selected from each bin to ensure representation across the full data range. Data points were plotted with slight horizontal jitter (width = 0.2) to avoid overplotting. Statistical significance was assessed using linear mixed-effects models with patients as a random effect.

### Model-free analyses

We adopted some widely used model-free measures, including win-stay and lose-shift^42^ as the direct measurement for this learning task.

#### Win-stay.

Win-stay is the percentage of times that the choice in trial t-1 was repeated on trial t following a reward.

#### Lose-switch.

In contrast, the lose-switch equals the percentage of trials that the choice was shifted or changed when the outcome of trial t-1 was non-reward. Model-free results can be found in Table S2.

### Kalman filter

The Kalman filter (KF) model has been widely applied in psychology and neuroscience to study various aspects of learning and decision-making^43,44^.

In the Kalman filter model for a multi-armed bandit task, *process noise*, and *observation noise* refer to two distinct sources of uncertainty that affect the learning and decision-making process.

Process noise represents the uncertainty in the evolution of the hidden state (reward mean) over time. It accounts for how the true state evolves from one point in time to the next. In mathematical terms, process noise is part of the state transition equation in the Kalman Filter:

$$x_{t}=x_{t-1}+e_{t}$$

$x_{t}$ is the state at time t

$e_{t}$ is the process noise t, which is assumed to be drawn from a normal distribution with zeros mean and **process noise variance** v. Where the $e_{t}\sim N(0,v)$.

The process noise captures the idea that the reward means for each arm can change from one trial to the next, even in the absence of any observations. A higher process noise variance v indicates a more volatile environment, where the reward means are expected to change more rapidly.

In contrast, **observation noise** represents the uncertainty in the observed rewards, given the current hidden state (reward mean). Which is assumed to be Gaussian with zero mean and a fixed variance $\sigma^{2}$.

The observation noise captures the idea that the observed rewards are noisy and can deviate from the true reward mean due to random fluctuations or measurement errors. A higher measurement noise variance indicates a more stochastic environment, where the observed rewards are less reliable and informative about the underlying reward means.

The Kalman Filter operates optimally when the statistical properties of the process noise and the measurement noise are accurately known.

When observation noise variance $\left(\sigma^{2}\right)$ is high relative to the process noise variance (v), the Kalman gain will be small, and the model will rely more on its prior beliefs and less on noisy observations. Conversely, when the observation noise variance (v), is high relative to the process noise variance ($\sigma^{2}$), the Kalman gain will be large, and the model will update its beliefs more strongly based on the observed rewards.

### Extended Kalman filter for three-armed bandit task

The Kalman filter model can be extended to capture the effects of both volatility and stochasticity in a multi-armed bandit task^17,45^.

In the current study, process noise variance (v) and observation noise variance ($\sigma^{2}$) represent volatility and stochasticity, respectively.

A traditional assumption of the Kalman filter is that the process noise variance, v, as well as the observation noise variance, $\sigma^{2}$ are constant.

Reward means update:

$$m_{t}=m_{t-1}+k_{t}\left(O_{t}-m_{t-1}\right)$$

Where $m_{t}$ is the estimated mean or value of the chosen arm at time t and $O_{t}$ is the observed reward at time t.

The mean update is driven by the prediction error, which is the difference between the observed reward and the previous estimate.

Kalman gain is defined as:

$$k_{t}=\left(w_{t-1}+v\right)/\left(w_{t-1}+v+\sigma^{2}\right)$$

Here, $k_{t}$ represents the Kalman gain or learning rate, which adjusts the weight given to new information based on the relative uncertainty of the prior estimate ($w_{t-1}$) and the total noise $\left(v+\sigma^{2}\right)$. When the stochasticity $\left(\sigma^{2}\right)$ is high relative to the volatility (v), the Kalman gain (learning rate) will be small, and the model will rely more on its prior beliefs and less on the observations. Conversely, when the volatility (v), is high relative to the stochasticity ($\sigma^{2}$), the Kalman gain (learning rate) will be large, and the model will update its beliefs more strongly based on the observed rewards.

Variance update equation:

$$w_{t}=\left(1-k_{t}\right)\left(w_{t-1}+v\right)$$

This equation updates the posterior variance $\left(w_{t}\right)$, which represents the estimate’s uncertainty after observing $O_{t}$.

### Volatile kalman filter for three-armed bandit task

The key difference between a standard Kalman filter and a volatile Kalman filter (VKF) is the variance of the process noise, a stochastic variable that changes with time. In other words, the VKF introduces parameters to handle the volatility in the process noise. Specifically, it allows the process noise variance v to vary with the observed prediction errors, reflecting changes in environmental volatility.

Our approach here is essentially the same as that taken by Piray and Daw (^17,45^. Here, we briefly described the model details as follows.

Kalman gain:

$$k_{t}=\left(w_{t}+v_{t-1}\right)/\left(w_{t-1}+v_{t-1}+\sigma^{2}\right)$$

where W is a noise parameter specific to binary observations. $\sigma^{2}$ capture the observation noise.

Update for the reward means:

$$m_{t}=m_{t-1}+k_{t}\left(O_{t}-m_{t-1}\right)$$

Update for posterior variance $w_{t}$:

$$w_{t}=\left(1-k_{t}\right)\left(w_{t-1}+v_{t-1}\right)w_{t-1,t}=\left(1-k_{t}\right)w_{t-1}$$

Update for volatility:

$$v_{t}=v_{t-1}+\lambda ({\left(m_{t}-m_{t-1}\right)}^{2}+w_{t-1}+w_{t}-2w_{t-1,t}-v_{t-1})$$

The model has three free parameters:

Where the λ represents the volatility learning rate, which is constrained to the unit range [0,1], determines how quickly volatility estimates update

### Soft-max choice-probability function for (volatile) kalman filter models

Decisions were modeled using a soft-max choice-probability function in which the probability of selecting a particular bandit i depends on its utility.

In the first soft-max function, we only included decision weights $\beta_{V}$ for expected-value.

The probability P of selecting bandit i in trial t was modeled as: $P_{i}(t)=\frac{e^{\beta_{V^{*}}V_{i}(t)}}{\sum_{j}\;\;e^{\beta_{V^{*}}V_{j}(t)}}$

In the second response model, we only included decision weights $\beta_{U}$ for outcome uncertainty to estimate the probability of choosing bandit i. So the probability P of selecting bandit i in trial t was modeled as:

$$P_{i}\left(t\right)=\frac{e^{\beta_{U}*U_{i}(t)}}{\sum_{j}\;e^{\beta_{U}*U_{j}(t)}}$$

We included the third response model’s decision weights for both expected value and outcome-uncertainty. So the probability P of selecting bandit i in trial t was modeled as:

$$P_{i}\left(t\right)=\frac{e^{\beta_{V}*V_{i}(t){+}^{\beta_{U}*U_{i}(t)}}}{\sum_{j}\;e^{\beta_{V}*V_{j}(t){+}^{\beta_{U}*U_{j}(t)}}}$$

### The definition for relative value and relative uncertainty

For each trial t, we computed the relative value (RV) and relative uncertainty (RU) of the chosen option compared to the unchosen options. Relative value was defined as the ratio of the chosen option’s estimated mean reward probability to the sum of all options’ value:

$$\text{RV}(t)=\frac{m_{t,\text{chosen}}}{\sum \;m_{t,\text{all}\;\text{options}}}$$

where $m_{t,\text{chosen}}$ is the estimated mean of the chosen option and $\sum \;m_{t,\text{all}\;\text{options}}$ represents the sum of estimated means across all options.

Similarly, relative uncertainty was calculated as the ratio of the chosen option’s posterior variance to the sum of all posterior variances:

$$\text{RU}\left(t\right)=\frac{W_{t,\text{chosen}}}{\sum \;W_{t,\text{all}\;\text{options}}}$$

where $W_{t,\text{chosen}}$ is the posterior variance of the chosen option and $\sum \;\;W_{t,\text{all}\;\text{options}}$ is the sum of posterior variances across all options.

### Rescorla-Wagner models

We also fitted the data to the classical Rescorla-Wagner model. Successful adaptation in a dynamic situation requires the appropriate feedback-based learning process where individuals integrate the feedback (reward or non-reward) into the stimulus-outcome association^46^. The basic reinforcement learning model, the Rescorla-Wagner model can address this process well. So the first model (RW1) was defined as:

$$v_{t}=v_{t-1}+a\times \left(R_{t-1}-v_{t-1}\right)$$

where $v_{t}$ is the value of the option on trial t.

a represents the general learning rate from feedback.

To verify whether participants employed distinct or shared computational responses to positive and negative feedback, we built another model with two learning rates, one for positive feedback and the other for negative feedback^42^. This model (RW2) can be defined as:

$$v_{t}=v_{t-1}+\alpha^{\text{pos}}\times \left(R_{t-1}-v_{t-1}\right),\text{positive}\;\text{feedback}$$

$$v_{t}=v_{t-1}+\alpha^{\text{neg}}\times \left(R_{t-1}-v_{t-1}\right),\text{negative}\;\text{feedback}$$

Where $v_{t}$ is the value of the option on trial $t.\alpha^{pos}$ and $\alpha^{neg}$ represent the learning rates from positive and negative feedback, respectively.

For these two models, $R_{t-1}\in \{0,1\}$ represents the feedback received in response to participants’ choice on trial t-1. And $R_{t-1}-v_{t-1}$ represents prediction error in trial t-1.

### Soft-max choice-probability function for Rescorla-Wagner models

We used a softmax choice function to map the value into choice. The softmax function for these four models can be defined as:

$$P_{i}\left(t\right)=\frac{e^{\beta_{V}*V_{i}(t)}}{\sum_{j}\;e^{\beta_{V}*V_{j}(t)}}$$

Where the $\beta_{V}$ represents the inverse temperature with choice value.

### Model fitting and comparison

Hierarchical Bayesian inference (HBI) is a powerful method for model fitting and comparison in group studies^19^. Unlike traditional approaches such as maximum likelihood estimation (MLE) or maximum a posteriori (MAP) estimation, which fit models to each subject independently, HBI simultaneously fits models to all subjects while constraining individual fits based on group-level statistics (i.e., empirical priors). This approach yields more robust and reliable parameter estimates, particularly when individual subject data is noisy or limited.

In our study, we employed HBI to fit models to choice data. The method quantifies group-level mean parameters and their corresponding hierarchical errors. To ensure that parameter estimates remain within appropriate bounds during the fitting process, we used the sigmoid function to transform parameters bounded in the unit range or with an upper bound and the exponential function to transform parameters bounded to positive values. The initial parameters of all models were obtained using a MAP procedure, with the initial prior mean and variance for all parameters set to 0 and 6.25, respectively, based on previous research^17^. This initial variance allows parameters to vary widely without substantial influence from the prior.

For model comparison, we used Bayesian model selection^47^, specifically employing the exceedance probability (XP) to select the winning model. The XP quantifies the probability that a given model is more frequent in the population than all other models under consideration while accounting for the possibility that the observed differences in model evidence may be due to chance. The model with the highest XP is selected as the winning model. The detailed results of our model comparison, including XP and BIC values for all models, can be found in Table S4.

### Model validation

We have conducted parameter recovery analysis that strictly follows the standard procedure described in the original methodology paper of VKF^17^ (page 21/26). We report the details in Supplementary Notes1. The recovery analysis revealed strong correlations between the true and recovered parameters (see Fig.S1)

### Medical imaging and processing

Pre-operative T1 MRI images without contrast were segmented and processed using *BrainSuite21a* (https://brainsuite.org/). Segmented MRI images and post-operative CT images were imported and aligned in Brainstorm 3 using the SPM12 (Statistical Parametric Mapping) (https://www.fil.ion.ucl.ac.uk/spm/). Electrode positions were then manually labeled using the post-operative CT images. Electrode coordinates were then exported to MNI coordinates and MRI space within Brainstorm.

### Electrode localization

To determine the brain area labels for each electrode, we jittered MNI coordinates 3 mm in all directions to account for co-registration errors, human errors, and inter-participant brain variability. Brain area labels were obtained from two brain atlases: Biolmage Suite Web (https://bioimagesuiteweb.github.io/webapp/) which provided gray matter Broadman area labels, and FAST (FMRIB’s Automated Segmentation Tool) (https://fsl.fmrib.ox.ac.uk/fsl/fslwiki/FAST) which provided Gray Matter, White Matter, and CSF labels. The jittering provided probabilistic electrode locations of which the top three labels were saved.

### Intracranial EEG collection and preprocessing

Intracranial signals were recorded from sEEG electrodes using a Neuralynx Atlas System at an 8 kHz sampling rate and subsequently downsampled to 500Hz for processing. Signal preprocessing consisted of several steps. (1) Signals were filtered between 1 Hz and 500 Hz using Fieldtrip and custom written scripts. (2) Line noise was removed using spectral interpolation at 60 Hz and its harmonics up to 300 Hz^48^. (3) Bad channels were removed using the ABCD algorithm^49^ which identified channels with abnormal power spectra in the 30 to 250 Hz range. (4) Bipolar re-referencing^50–52^ on nearest neighbors on the same electrode was used to further reduce noise, artifacts, and common signals.

### Neural signals alignment

Neural signals were aligned to behavioral events using TTL (Transistor-Transistor Logic) pulses. TTL signals were simultaneously recorded with neural data and task events to ensure precise temporal alignment. We performed post-hoc timing analysis to verify the temporal alignment between neural recordings and behavioral events, confirming timing consistency within 2 ms or less. For event-aligned analyses, neural signals were temporally aligned using TTL timestamps, with additional behavioral parameters (e.g., stimulus information) extracted from the behavioral data stream.

### Electrode selection and definition for dIPFC

We only used the top brain area labels from both of the electrodes used to calculate the bipolar channel montage.The dIPFC channels were selected from bipolar channels in which either electrode was located in BA 46.

### Region of interest definition for dmPFC

Following recent theoretical frameworks^53^, we combined recordings from the dorsomedial prefrontal cortex (dmPFC) and dorsal anterior cingulate cortex (dACC) into a single functional region of interest, referred to as dmPFC. This approach acknowledges the anatomical and functional overlap between these regions, which form a functional cluster commonly observed in neuroimaging studies. The dmPFC was defined as the medial portion of Brodmann areas 8 and 9, while the dACC was defined as the dorsal portion of areas 24 and 32, anterior to the vertical commissure anterior (VCA) line^54^. Given that this brain area represents a functional cluster overlapping the cingulate gyrus and frontal lobe without clear anatomical boundaries, we designated it as the dmPFC area to denote its general anatomical location. This combined approach aligns with mounting evidence that the dmPFC forms an integrated neural hub for higher-order cognitive processes^55^.

### Time-frequency analyses

Time-frequency decomposition was performed using Complex Morlet Wavelets based on established methods^56^. The wavelet was defined as a complex sine wave tapered by a Gaussian window for each frequency. To optimize the trade-off between temporal and frequency precision across different frequency bands, we implemented a frequency-dependent cycle width that logarithmically increased from 4 cycles at the lowest frequency (4 Hz) to 9 cycles at the highest frequency (150 Hz). Signal power and phase were calculated across 50 logarithmically spaced frequency bins from 4 Hz to 150 Hz.

For the full feedback stage analysis, spectral data were epoched from 500 ms before to 2000 ms after outcome onset to capture the full feedback duration (1500 ms). For the decision stage, epochs were extracted from 1000 ms before to 1000 ms after selection onset. The epoched data were organized into three-dimensional matrices (time points× frequency bins × trials) with corresponding event information (e.g., reward vs. non-reward, stay vs. switch) stored in parallel MATLAB tables. After combining data across all sessions and patients, the final matrices comprised 1250 time points × 50 frequency bins × 99,387 trials for dIPFC, and 1250 × 50 × 56,140 for dmPFC in full feedback stage; and 1000 time points × 50 frequency bins × 99,387 trials for dIPFC, 1000 × 50 × 56,140 for dmPFC in decision stage

### Linear mixed effects models (LMEs)

Neural activity was analyzed using linear mixed effects models (LMEs) with hierarchical random effects to account for multiple channels recorded from each patient. Our analysis proceeded in two stages: first, a comprehensive time-frequency analysis to identify relevant frequency bands, followed by targeted analysis of specific bands (Hilbert transform) of interest.

Initially, we applied LMEs to the full time-frequency data to identify which frequency bands were involved in processing feedback and decisions. For all models, spectral power (referred to as ‘erd’) served as the dependent variable, with patient ID and channel nested within patient ID included as random intercepts.

For the post-feedback stage, we built the following LMEs:

1. To examine neural differentiation of feedback outcomes: *erd ~ reward + previous trial feedback + next trial decision + (1|patientID) + (1|channelID:patientID)* where reward distinguishes between reward and non-reward outcomes
2. To capture the PE representation, *erd ~ PE + value + uncertainty + (1|patientID) + (1|channelID:patientID)* where PE reflect the prediction error of chosen option, value, uncertainty reflect updates following the current feedback for the chosen option
3. To capture value and uncertainty representation: *erd ~ relative value + relative uncertainty + (1|patientID) + (1|channelID:patientID)* where relative value and uncertainty reflect updates following the current feedback

For the pre-selection stage, we constructed:

1. To examine neural correlates of choice behavior: *erd ~ decision + previous trial feedback + (1|patientID) + (1|channelID:patientID*) where decision distinguishes between stay and switch choices
2. To capture the neural representation for PE, *erd ~ PE + value(of choice) + uncertainty(of choice) +(1|patientID) + (1|channelID:patientID)*
3. To capture value and uncertainty influence: *erd ~ relative value + relative uncertainty + (1|patientID) + (1|channelID:patientID)*

All binary variables were coded as follows:

decision (*next trial decision*): stay = 1, switch = 0

feedback-type: reward = 1, non-reward = 0

Importantly, the prediction error, relative value and uncertainty terms in both post-feedback and pre-selection models are identical, as both stages occur before the next choice (stay/switch) is made. These variables reflect the updated estimates following the most recent outcome, which remain constant until the next decision is executed.

The nested random effects structure (channelID:patientID) accounts for the hierarchical nature of our data, which included multiple channels recorded from each patient.

### 2D Cluster-based permutation test

Next, we performed 2D cluster-based permutation tests (CBPT) with linear mixed effects models (LMEs) to determine if the spectral data correlated with specific task variables (e.g., reward vs. non-reward, stay vs. switch)^57^. We used a minimum cluster size of 4 Hz × 80 ms (i.e., 320 Hz×ms pixels). The significant threshold, α, was set at 0.05 but divided by the number of fixed effects. A cluster is considered significant if the cluster-level statistic for that cluster exceeds the 100*(1-α)-percentile of the shuffled sum (*t-statistic*^2^) distribution from the largest cluster. To be more specific, for each identified cluster, we computed a cluster-level statistic by summing the squared t-statistics of all time-frequency points within that cluster. Statistical significance was assessed through a permutation procedure with 5000 iterations. In each permutation, trial labels were randomly shuffled while maintaining the hierarchical data structure, LMEs were refitted, and the largest cluster-level statistic was recorded to build a null distribution. A cluster from the original data was considered significant if its cluster-level statistic exceeded the 95th percentile of this null distribution, effectively controlling for multiple comparisons while maintaining sensitivity to detect temporally and spectrally extended effects.

### High-gamma and theta-band focused analysis

Band-limited analysis in the high-gamma band (70–150 Hz) and theta-band (4–9 Hz) was carried out using the Hilbert Analytic Amplitude (HAA) method^58,59^. For each frequency band, the continuous neural signal was divided into eight logarithmically spaced sub-bands. Each sub-band was then filtered using a Gabor filter implemented with a Gaussian envelope in both the time and frequency domains to achieve optimal time–frequency resolution. The filter is defined as:

$$g(t)=Ae^{-s_{0}{\left(t-t_{0}\right)}^{2}}e^{2\pi iv_{0}\left(t-t_{0}\right)}$$

Where:

$t_{0}$ is the center time (0)

$v_{0}$ is the center frequency

$s_{0}$ is the duration parameter, defined as:

$$s_{0}=ln\left(\frac{2\text{ln}\;\left(2\right)}{\text{fb}w^{2}\pi {v_{0}}^{2}}\right)$$

where *fbw* is the fractional bandwidth (0.25).

The amplitude normalization constant A is defined as

$$A=2^{1/4}e^{-s_{0}/4}$$

Which ensures that the Gabor filter has unit energy (i.e., unit norm) so that its contribution remains independent of its absolute amplitude. The standard deviations of the Gaussian envelope in the time and frequency domains were defined as:

$$\sigma_{t}=\sqrt{\frac{e^{s_{0}}}{4\pi }}$$

$$\sigma_{f}=\sqrt{\frac{e^{-s_{0}}}{4\pi }}$$

These parameters guarantee a balanced trade-off between temporal precision and frequency selectivity. Following filtering, the Hilbert transform (implemented via MATLAB’s *hilbert* function) was applied to the filtered signal to obtain the analytic signal, and its absolute value was computed to yield the instantaneous power (analytic amplitude). Finally, the analytic amplitudes across all eight sub-bands were averaged to generate a single time series representing the overall band-specific activity. This approach provides a more stable estimate of broadband power compared to using a single wide bandpass filter, as it accounts for the non-uniform distribution of power across frequencies while preserving physiologically relevant temporal resolution. Subsequent statistical analyses, including linear mixed effects models (see above) and cluster-based permutation tests (see below) were applied to these band-limited time series to assess task-related modulations in neural activity.

### Cross-frequency phase-amplitude coupling analysis

To investigate functional interactions between dIPFC and dmPFC, we examined cross-frequency coupling between high-frequency power (30–150 Hz) in dmPFC and theta phase (4–14 Hz) in dIPFC. We performed exploratory phase-amplitude coupling (PAC) analysis separately for post-feedback and pre-selection stages to identify optimal frequency pairs showing maximal coupling. Signal power and phase were calculated across 50 logarithmically spaced frequency bins from 4 Hz to 150 Hz, resulting in an 18 × 22 analysis grid (18 phase bins below 15 Hz and 22 frequency bins above 30 Hz). For each frequency pair in the grid, we quantified PAC using the modulation index^60^.

$$\text{PAC}=\left|n^{-1}\sum_{t=1}^{n}\;a_{t}e^{i\emptyset_{t}}\right|$$

where t is the time point, $a_{t}$ is the power at a given frequency, $\emptyset_{t}$ is the phase angle (in radians) at another frequency, i is the imaginary operator, and n is the total number of time points.

For each frequency pair, we generated a null distribution by randomly shifting the power time series while keeping the phase time series fixed, computing the PAC value for each iteration. This procedure was repeated 5000 times to create a distribution of PAC values expected under the null hypothesis of no temporal relationship between phase and power.

The observed PAC value was then compared to this null distribution by converting it to a standardized Z-score, calculated by subtracting the mean and dividing by the standard deviation of the null distribution.

The exploratory analysis revealed maximal coupling between 9 Hz phase in dIPFC and 112 Hz power in dmPFC in post-feedback stage, and 4 Hz phase in dIPFC and 42 Hz power in dmPFC for the decision stage (Fig. S5).

This optimal frequency pair was then used for subsequent analyses of condition-specific effects. Statistical analysis was performed as described above using the same models.

### Power-based connectivity analysis

We examined power-based connectivity between the dorsolateral prefrontal cortex (dIPFC) and the dorsomedial prefrontal cortex (dmPFC) using amplitude-amplitude coupling (AAC) measures. We conducted two connectivity analyses.

#### Within-frequency theta band coupling (4–9 Hz).

For both the dIPFC and dmPFC, the neural signals were first band-limited to the theta frequency range (4–9 Hz) using our Hilbert Analytic Amplitude (HAA) approach. In brief, the raw signals were filtered with a Gabor filter tailored to the theta band, and the Hilbert transform was applied to obtain the analytic signal. The absolute value of the analytic signal provided the instantaneous power (analytic amplitude). To quantify the functional coupling, we computed the Pearson correlation coefficient between the theta-band HAA time series from dIPFC and dmPFC. This correlation reflects the degree to which fluctuations in theta power are synchronized between the two regions, thereby serving as an index of their functional connectivity.

#### Across-frequency coupling (dmPFC high-gamma and dIPFC theta).

We also investigated cross-frequency interactions by examining the coupling between high gamma activity (70–150 Hz) in the dmPFC and theta activity (4–9 Hz) in the dIPFC. The analytic amplitude time series for the dmPFC high gamma band was computed in a similar manner as described above, while the theta band analytic amplitude was extracted from the dIPFC. The Pearson correlation coefficient was then calculated between these two time series to assess cross-frequency amplitude-amplitude coupling.
